# Influence of Death from Circulatory Diseases on Life Expectancy at Birth in Japan

**DOI:** 10.2188/jea.12.450

**Published:** 2007-11-30

**Authors:** Tomoyuki Watanabe, Masako Omori, Hiromi Fukuda, Hiroki Takada, Masaru Miyao, Isao Ohsawa, Yoshiharu Oshida, Yuzo Sato, Toshihiko Hasegawa

**Affiliations:** 1 Graduate School of Medicine, Nagoya University.; 2 Graduate School of Science, Nagoya University.; 3 Information Technology Center, Nagoya University.; 4 Research Center of Health, Physical Fitness and Sports, Nagoya University.; 5 National Institute of Public Health.

**Keywords:** life expectancy, circulatory diseases, mortality

## Abstract

This study aims to evaluate the contribution of the change in circulatory diseases mortality to the life expectancy at birth observed during the years 1955-1995 in Japan. We used data on the population and the number of deaths by cause, age, and sex in 1955, 1965, 1975, 1985, and 1995. The contribution of different ages and causes of death to the change in life expectancy were examined with the method developed by Pollard. We found that the reduction in circulatory diseases mortality contributed to the improvements in life expectancy for both sexes during the decade 1975-1985. Much of this was due to the decrease in cerebrovascular disease. In the years 1985-1995, however, the contribution of cerebrovascular disease decreased in both sexes, while that of heart disease grew to become the largest of any single condition. By age, the contribution of all circulatory diseases increased among the elderly in recent years. The contribution of the change in circulatory diseases mortality to the life expectancy at birth has increased in recent years but seems to have reached a plateau. The weight against improvements in life expectancy in middle-aged people has shown little change, so that reducing the mortality rate in middle-aged people is now a major issue.

Since 1984, life expectancy at birth among the Japanese has been the longest in the world at any time in history.^1^ This marked change in life expectancy at birth has been accompanied by a health transition that is represented by a dramatic decrease in infectious diseases such as tuberculosis. The recent increase in Japanese longevity is due partly to effective countermeasures for so-called “lifestyle-related diseases,” which include arteriosclerotic diseases like cerebrovascular disease. However, circulatory diseases remain one of the major causes of death in Japan. Cerebrovascular disease had long been the leading cause of death, until it began decreasing greatly in the latter 1960s. In 1981, it was taken over by malignant neoplasm as the second leading cause of death. Since then, it has continued to decline, and today ranks as the third leading cause of death. Heart disease, formerly third among the causes of death, became the number two cause of death in 1985. It showed temporary decline in recent years, but has now regained its former high position.^2^

Various indicators are used to analyze the structure of causes of death. However, there have been few studies analyzing the contribution of each to life expectancy at birth due to the changes in mortality by each cause of death.

In this study, we examined the contribution of different age groups and causes of death, especially circulatory diseases, to the change in life expectancy at birth.

## MATERIALS AND METHODS

The figures used in the present study for population size and the number of deaths by cause, age (5-year groups), and sex in 1955, 1965, 1975, 1985, and 1995 are based on the vital statistics of Japan.^2^^-^^4^ Life expectancies were estimated from the complete life tables in 1955, 1965, 1975, 1985, and 1995.^1^ We analyzed the changes in cause of death for each of the following 10-year periods:1955-1965, 1965-1975, 1975-1985, and 1985-1995. The diseases examined were all causes, circulatory diseases (including cerebrovascular disease), circulatory diseases (excluding cerebrovascular disease), cerebrovascular disease (CVD), ischemic heart disease (IHD), and heart disease (except for IHD). The causes of death were classified according to the International Classification of Diseases and Causes of Death as shown in Table 1. For age comparison, we classified the data into 5 groups according to subject age as follows: 0-14, 15-44, 45-64, 65-74, and 75 years or over.

**Table 1.** Classification of causes of death by circulatory diseases

	Calendar year (ICD version)				

Causes	1955	1965	1975	1985	1995
	(ICD-6)	(ICD-7)	(ICD-8)	(ICD-9)	(ICD-10)
Circulatory diseases (including cerebrovascular disease*)	330-468	330-468	390-458	390-459	I00-I99

Circulatory diseases (excluding cerebrovascular disease*)	400-468	400-468	390-404, 420-429, 440-458	390-405, 415-429, 440-459	I00-I15, I26-I52, I70-I99

Cerebrovascular disease (CVD)	330-334	330-334	430-438	430-438	I60-I69

Heart disease (except for ischemic heart disease)	400-416, 421-434	400-416, 421-434	390-398, 420-429	390-398, 415-417	I00-I09, I26-I52, I95-I99

Ischemic heart disease (IHD)	420	420	410-414	410-414	I20-I25

* Since cerebrovascular disease is not classified in cardiovascular diseases but in vascular lesions affecting central nervous system in 1955 (ICD-6) and 1965 (ICD-7), we divided cardiovascular disease into including cerebrovascular disease and excluding cerebrovascular disease.

When a life table was applied at the two time points t_1_ and t_2_, the difference in life expectancy at birth e_0_^2^-e_0_^1^ depended on the changes in mortality for each age group at the two time points. Thus, using the method of Pollard,^5^^-^^7^ we were able to evaluate how the changes in mortality from different causes of death produced differences in life expectancy at birth at these two times. That is, we weighted changes in life expectancy against changes in mortality by circulatory diseases, and calculated this as contributing year.

The following formula can be used to analyze change in life expectancy at birth according to mortality trends by age and cause:

$$\begin{array}{rl} & e_{0}{}^{2}-e_{0}{}^{1}≅\sum_{\text{i}}{(}_{1}m_{0}{}^{\text{(i)1}}-{}_{1}m_{0}{}^{\text{(i)2}})w_{0}+4\sum_{\text{i}}{(}_{4}m_{1}{}^{\text{(i)1}}-{}_{4}m_{1}{}^{\text{(i)2}})w_{2}\\ & \;+5\sum_{\text{i}}{(}_{5}m_{5}{}^{\text{(i)1}}-{}_{5}m_{5}{}^{\text{(i)2}})w_{7.5}+5\sum_{\text{i}}{(}_{5}m_{10}{}^{\text{(i)1}}-{}_{5}m_{10}{}^{\text{(i)2}})w_{12.5}+\cdots \end{array}$$

$$\text{with }w_{\text{t}}=1/2{(}_{\text{t}}p_{0}{}^{2}e_{\text{t}}{}^{1}+{}_{\text{t}}p_{0}{}^{1}e_{\text{t}}{}^{2}),\;{}_{\text{n}}m_{\text{x}}{}^{\left(\text{i}\right)}={}_{\text{n}}m_{\text{x}}{(}_{\text{n}}D_{\text{x}}{}^{\left(\text{i}\right)}{/}_{\text{n}}D_{\text{x}})$$

where the suffixes 1 and 2 on the life table functions and mortality rate refer to times 1 and 2; _t_p_x_^1^, _t_p_x_^2^ is the probability of survival t years from x at times 1 and 2, respectively; e_x_^1^, e_x_^2^ is the expectation of life at age x; and _n_m_x_^(i)1^, _n_m_x_^(i)2^ is the central mortality rate for cause i in an age interval (x, x+n-1) at times 1 and 2, respectively. _n_D_x_ is the number of deaths from all causes in an age interval (x, x+n-1), and _n_D_x_^(i)^ is the number of deaths by cause i in an age interval (x, x+n-1). Finally, w_t_ is the weight function.

A positive contribution indicates that a mortality reduction in the relevant age group contributes to an increase in life expectancy at birth, whereas a negative contribution indicates that a mortality increase contributes to a reduction in life expectancy at birth.

For this paper, we estimated the contributions of circulatory diseases to changes in life expectancy by age group and cause of death.

## RESULTS

Tables 2 and 3 show the contributions of mortality changes by all causes and circulatory diseases to the life expectancy at birth during the years 1955-1995 for male and female, respectively.

**Table 2.** Contributions of mortality changes by circulatory diseases to the life expectancy at birth between 1955 and 1995 (Male)

Age (year)	Calendar year			

	1955-1965	1965-1975	1975-1985	1985-1995
All causes				
0-14	2.289	0.944	0.587	0.146
15-44	1.088	0.582	0.491	0.239
45-64	0.640	1.085	0.573	0.423
65-74	0.132	0.809	0.810	0.253
75-	-0.104	0.511	0.596	0.526
Total	4.044	3.931	3.057	1.587
Circulatory diseases (including cerebrovascular disease)				
0-14	0.018	0.003	0.000	0.009
15-44	0.033	0.024	0.083	0.068
45-64	0.071	0.530	0.392	0.230
65-74	-0.124	0.490	0.583	0.285
75-	-0.262	0.137	0.522	0.529
Total (%*)	-0.264 (-6.5)	1.184 (30.1)	1.581 (51.7)	1.121 (70.6)
Circulatory diseases (excluding cerebrovascular disease)				
0-14	0.020	0.004	-0.002	0.008
15-44	0.053	0.008	0.018	0.043
45-64	0.017	0.079	0.035	0.117
65-74	-0.039	0.096	0.092	0.149
75-	-0.116	0.031	0.079	0.337
Total (%)	-0.066 (-1.6)	0.217 (5.5)	0.221 (7.2)	0.654 (41.2)
Cerebrovascular disease (CVD)				
0-14	-0.001	-0.001	0.003	0.001
15-44	-0.019	0.017	0.065	0.025
45-64	0.054	0.451	0.357	0.112
65-74	-0.085	0.395	0.491	0.136
75-	-0.146	0.106	0.444	0.192
Total (%)	-0.197 (-4.9)	0.967 (24.6)	1.360 (44.5)	0.467 (29.4)
Ischemic heart disease (IHD)				
0-14	0.000	0.000	0.000	0.000
15-44	0.001	0.010	0.014	-0.014
45-64	-0.069	0.029	0.050	-0.016
65-74	-0.079	-0.005	0.064	0.010
75-	-0.055	-0.036	0.045	-0.005
Total (%)	-0.201 (-5.0)	-0.001 (0.0)	0.172 (5.6)	-0.025 (-1.6)
Heart disease (except for ischemic heart disease)				
0-14	0.018	0.004	-0.002	0.006
15-44	0.048	0.003	-0.004	0.056
45-64	0.071	0.055	-0.053	0.129
65-74	0.033	0.088	-0.023	0.127
75-	-0.059	0.080	-0.049	0.285
Total (%)	0.111 (2.7)	0.230 (5.9)	-0.131 (-4.3)	0.604 (38.0)

Values and percentages in this column may not add up to exactly total number and 100% because of rounding, respectively

* The proportion of the contribution from changes in circulatory diseases mortality against changes in life expectancy from all causes of death

**Table 3.** Contributions of mortality changes by circulatory diseases to the life expectancy at birth between 1955 and 1995 (Female)

Age (year)	Calendar year			

	1955-1965	1965-1975	1975-1985	1985-1995
All causes				
0-14	2.624	0.786	0.457	0.120
15-44	1.410	0.546	0.353	0.133
45-64	0.914	0.950	0.708	0.304
65-74	0.383	0.834	0.860	0.483
75-	-0.068	0.797	1.236	1.306
Total	5.262	3.913	3.614	2.346
Circulatory diseases (including cerebrovascular disease)				
0-14	0.022	0.004	0.003	0.009
15-44	0.165	0.089	0.053	0.044
45-64	0.303	0.478	0.341	0.187
65-74	-0.013	0.499	0.566	0.350
75-	-0.385	0.110	0.827	1.031
Total (%*)	0.091 (1.7)	1.180 (30.2)	1.789 (49.5)	1.620 (69.1)
Circulatory diseases (excluding cerebrovascular disease)				
0-14	0.023	0.006	-0.001	0.008
15-44	0.135	0.072	0.029	0.031
45-64	0.065	0.134	0.093	0.092
65-74	-0.017	0.105	0.115	0.187
75-	-0.184	-0.013	0.202	0.625
Total (%)	0.022 (0.4)	0.303 (7.7)	0.438 (12.1)	0.943 (40.2)
Cerebrovascular disease (CVD)				
0-14	-0.001	-0.001	0.004	0.001
15-44	0.030	0.017	0.024	0.013
45-64	0.238	0.344	0.247	0.095
65-74	0.004	0.394	0.451	0.163
75-	-0.201	0.123	0.625	0.406
Total (%)	0.069 (1.3)	0.877 (22.4)	1.351 (37.4)	0.677 (28.9)
Ischemic heart disease (IHD)				
0-14	0.000	0.000	0.000	0.000
15-44	0.011	0.009	0.006	-0.005
45-64	-0.037	0.030	0.041	-0.002
65-74	-0.065	-0.007	0.062	0.026
75-	-0.072	-0.070	0.077	0.015
Total (%)	-0.163 (-3.1)	-0.037 (-1.0)	0.187 (5.2)	0.033 (1.4)
Heart disease (except for ischemic heart disease)				
0-14	0.021	0.005	-0.001	0.008
15-44	0.120	0.066	0.017	0.032
45-64	0.088	0.098	0.025	0.087
65-74	0.036	0.105	-0.008	0.143
75-	-0.111	0.105	-0.022	0.472
Total (%)	0.155 (2.9)	0.379 (9.7)	0.010 (0.3)	0.743 (31.7)

Values and percentages in this column may not add up to exactly total number and 100% because of rounding, respectively

* The proportion of the contribution from changes in circulatory diseases mortality against changes in life expectancy from all causes of death

### 1. All causes

The contribution to change in life expectancy from all causes of death showed a downward trend in both sexes. The improvement by all causes in the years 1985-1995 decreased more than half as large as that in the years 1955-1965. In the ten years from 1955 through 1965, the contribution of the young group aged 0-14 years was large (2.289 years), but afterward it decreased dramatically. Overall, the change in life expectancy has become smaller as the present day is approached. On the contrary, the contribution among 75 years or over age group showed an upward trend.

### 2. Circulatory diseases (including CVD)

The contribution to improvements in life expectancy from circulatory diseases, including CVD showed an upward trend in both sexes until the 1975-1985 period, during which it began to fall. In the 1955-1965 period, the contribution was negative among the elderly of both sexes, but afterward there was a positive contribution in the middle-aged and elderly. The contribution increased in those 75 years of age and over especially for female, but decreased in people aged 15-44 and 45-64 years.

### 3. Circulatory diseases (excluding CVD)

The contributing years for circulatory diseases excluding CVD showed a rising trend in both males and females, and a large contribution to improvements in life expectancy was seen in the years 1985-1995. This contributing year was 0.654 years for male, and 0.943 years for female. By age, most contribution was seen among the middle-aged and elderly. In particular, among the elderly 75 years of age and above, a negative contribution with a large weight was seen in the years 1955-1965 (male: -0.116 years and female: -0.184 years), but then in the years 1985-1995 it accounted for a large weight in improvements in life expectancy (male: 0.337 years and female: 0.625 years).

### 4. Cerebrovascular disease (CVD)

The weights accounted for by improvements in life expectancy for both sexes were large, especially from 1965 through 1985. CVD was a cause of reduced life expectancy for males in the years 1955-1965 (-0.197 years), but afterward increased so that it showed a large weight in improvements in life expectancy in the years 1975-1985 (1.360 years). In 1985-1995, however, the contribution became smaller. The trend in female was also similar to male. By age group, people in the 45-64, 65-74, and 75 years and older age groups accounted for the majority of the contribution to life expectancy in both sexes.

### 5. Ischemic heart disease (IHD)

The contributions of IHD to changes in life expectancy at birth in both sexes were small and broadly flat from 1955 through 1995. There was a large negative contribution in the years 1955-1965 (-0.201 years for male and -0.163 years for female), but this increased until the largest positive contribution was seen in the years 1975-1985 (0.172 years for male and 0.187 years for female). Afterwards, a decrease was seen and in 1985-1995 there was again a negative contribution in males. By age, the majority of the contribution was accounted for by the middle-aged and elderly. In the years 1955-1965 and 1965-1975, death in middle and old age was a factor shortening life expectancy.

### 6. Heart disease (excluding IHD)

Until 1985, the number of years contributed by heart disease other than IHD to change in life expectancy was small for both sexes. However, there was a much greater contribution from IHD for both males and females in the years 1985-1995 (male: 0.604 years and female: 0.743 years). By age, the contributions of those aged 65 years or more of both sexes were greater. In the years 1985-1995, the weight accounted for by those 75 years of age or more was particularly large (male: 0.285 years and female: 0.472 years).

### 7. Proportion of changes in life expectancy accounted for by all caused of death

The level of the contribution from changes in circulatory diseases mortality against changes in life expectancy from all causes of death in each 10-year period was also given in Tables 2 and 3. The proportion of improvements in life expectancy accounted for by decreased mortality from all circulatory diseases has increased with time until today. In the 1985-1995 period this accounted for about 70% of the total in both sexes. While the percentage of CVD decreased, that of heart disease increased.

## DISCUSSION

In Japan, the life expectancy at birth has been lengthening each year as a result of decreased mortality. However, the changes do not necessarily show uniform trends in all age groups, and mortality curves differ.^8^ Numerous studies have analyzed the structure of causes of death. However, few studies have investigated and analyzed the contributions to changes in life expectancy at birth by cause of death and age group,^8^^-^^11^ and there have been almost no such studies in Japan.

One method to estimate the contributions by cause of death is known as potential gains in life expectancy by complete elimination of a specific cause of death.^12^^,^^13^ However, the idea that one can completely eliminate certain causes of death is not realistic.^9^ It is more realistic to measure the contribution to an increase or a reduction in life expectancy at birth by the changes in different age groups and causes of death. This method, by indicating which diseases are being overcome and which have a higher morbidity and severity at which periods in time, is thought to be useful in that if offers a means of analyzing the types of changes in life expectancy at birth by age group.

In the present study we looked at which circulatory diseases affected changes in life expectancy in Japan, and the level of those effects, over the 40 years from 1955 through 1995.

The decreased mortality from circulatory diseases (including CVD) each year in the 75 year old and over population has had a great impact on the improvement in life expectancy. A particularly large increase has been seen in women. Nusselder et al. analyzed the contribution to improvements in life expectancy from decreased morbidity of various diseases, and reported that the decreased mortality from circulatory diseases among the elderly in recent years has had a large effect in improving life expectancy.^14^ There was little improvement in mortality in the 45-74 age group, so the contributions of this age group to improvements in life expectancy were small. It is characteristic of the Pollard method that changes at young ages have a larger impact on life expectancy than changes at advanced ages. Changes in mortality at younger ages tend to have a greater impact on changes in life expectancy. Despite this, from the considerable contribution of the elderly seen in recent years and the small contribution of the middle-aged group, we can see that while the decline in mortality from circulatory diseases among the elderly is marked, there has been little improvement in this mortality rate in the middle-aged group.

Considering circulatory diseases other than CVD, we see that the contribution to improvements in life expectancy is increasing year by year, with a particularly striking contribution in recent years. In the first decade covered in this study, 1955-1965, the 75 and over age group of both sexes had a shortened life expectancy, but in the years 1985-1995 this was the group with the greatest gains in life expectancy. In recent years the 65-74 group has also had a large impact on improvements in life expectancy in both males and females.

The mortality from CVD during the 20 years from 1965 through 1985 declined dramatically, and this contributed greatly to an expanded life expectancy at birth. Reports from many countries, including those of Southeast Asia,^15^^-^^17^ have described a declining mortality from CVD.^18^^-^^21^ The reason for this is unclear,^22^ but it is thought to be related to the decreased incidence of stroke.^23^^-^^25^ Conversely, there are also reports of an increasing incidence of stroke in some countries.^26^^,^^27^ In recent years, the decline in CVD has leveled, and its contribution to improvements in life expectancy has largely decreased. Until the mid-1980s, the decreasing mortality from CVD was a major factor contributing to improvements in life expectancy. In recent years, however, heart disease has taken its place, and has come to make the largest contribution among circulatory diseases. In particular, the declining contribution in those aged 45-74 years has in recent years pulled down the contribution of this age group for circulatory diseases overall.

IHD had no large impact on change in life expectancy in either sex. The contribution of reduced heart disease to improvement in life expectancy is mostly due to decreases in heart disease other than IHD. Mortality from IHD decreased in 1975-1985, but in other periods remained nearly unchanged. The IHD mortality in Japan has tended downward since 1970, and in 1985 became the lowest in all the industrialized countries.^18^^,^^28^ One of the reported causes of the decrease in IHD mortality in Japan is the decrease in hypertension.^29^^-^^31^ Conversely, in the 10 years of 1985-1995, IHD has shown a negative contribution in males and in recent years has increased slightly.

Japan adopted the International Statistical Classification of Diseases and Related Health Problem, Tenth Revision (ICD-10) in 1995.^32^ One of the aims in this was to correct the overdiagnosis of heart failure, and increase the reliability of mortality statistics by cause of death based on death certificates. This has had a large impact on mortality statistics, especially for death due to heart disease. A large decline has been seen in the recorded number of deaths from heart failure, and a dramatic increase in deaths from IHD.^33^ However, despite the various studies that have been conducted,^33^^-^^36^ no epidemiologic basis has been presented for whether the reliability of death certificates has improved with respect to the sudden increase in IHD. For the above reasons, this problem remains an issue for future study.

Although the absolute value of the contribution of circulatory diseases overall is moving downward, its proportion of overall contributions is increasing. This is because even though the contribution from reductions in circulatory diseases is decreasing, the contributions from other causes of death are also decreasing greatly, resulting in a declining contribution from all causes of death.

Thus, while mortality from circulatory diseases in Japan is decreasing year by year, they remain one of the major causes of death. Their contribution to changes in life expectancy has increased in recent years but seems to have reached a plateau, and the decline in mortality from CVD has grown smaller. Mortality from heart disease, on the other hand, has dropped significantly in recent years, with a particularly striking decline in mortality among the elderly. However, the weight against improvements in life expectancy in middle aged people has shown little change, so that reducing the mortality rate in middle aged people is now a major issue. The findings in our present analysis may have implications for practical decision making in setting up health goals and evaluating health promotion activities.
